# UBN2 promotes tumor progression via the Ras/MAPK pathway and predicts poor prognosis in colorectal cancer

**DOI:** 10.1186/s12935-019-0848-4

**Published:** 2019-05-10

**Authors:** Ya-li Zhao, Shen-Rong Zhong, Shi-Hong Zhang, Jia-Xin Bi, Zhi-Yuan Xiao, Shu-Yang Wang, Hong-Li Jiao, Dan Zhang, Jun-Feng Qiu, Ling-Jie Zhang, Cheng-Mei Huang, Xiao-Ling Chen, Yan-Qing Ding, Ya-Ping Ye, Li Liang, Wen-ting Liao

**Affiliations:** 1 Department of Pathology, Nanfang Hospital, Southern Medical University, Guangzhou, 510515 Guangdong China; 2 Department of Pathology, School of Basic Medical Sciences, Southern Medical University, Guangzhou, Guangdong China; 3 Guangdong Provincial Key Laboratory of Molecular Tumor Pathology, Guangzhou, Guangdong China; 4 Department of Laboratory Medicine, The First Affiliated Hospital of Sun Yat-sen University, Guangzhou, Guangdong China

**Keywords:** UBN2, Proliferation, Metastasis, Prognosis, Colorectal cancer, Ras/MAPK

## Abstract

**Background:**

Ubinuclein-2 (UBN2) is a nuclear protein that interacts with many transcription factors. The molecular role and mechanism of UBN2 in the development and progression of cancers, including colorectal cancer (CRC), is not well understood. The current study explored the role of UBN2 in the development and progression CRC.

**Methods:**

Oncomine network and The Cancer Genome Atlas (TCGA) database were downloaded and Gene Set Enrichment Analysis (GSEA) was performed to compare the UBN2′s expression between normal and tumor tissues, as well as the potential correlation of UBN2 expression with signaling pathways. Immunohistochemistry (IHC), qRT-PCR and Western blotting were performed to determine the expression of UBN2 in CRC tissues or cell lines. In vitro proliferation and invasion assays, and orthotopic mouse metastatic model were used to analyze the effect of UBN2 on the development and progression of CRC.

**Results:**

The analysis of UBN2 expression using Oncomine network showed that UBN2 was upregulated in CRC tissues compared to matched adjacent normal intestinal epithelial tissues. IHC, qRT-PCR and Western blotting confirmed that UBN2 expression is higher in CRC tissues compared with matched adjacent normal intestinal epithelial tissues. In addition, analyses of TCGA data revealed that high UBN2 expression was associated with advanced stages of lymph node metastasis, distant metastasis, and short survival time in CRC patients. IHC showed that high UBN2 expression is correlated with advanced stages of CRC. Moreover, UBN2 is highly expressed in the liver metastatic lesions. Furthermore, knockdown of UBN2 inhibited the growth, invasiveness and metastasis of CRC cells via regulation of the Ras/MAPK signaling pathway.

**Conclusion:**

The current study demonstrates that UBN2 promotes tumor progression in CRC. UBN2 may be used as a promising biomarker for predicting the prognosis of CRC patients.

**Electronic supplementary material:**

The online version of this article (10.1186/s12935-019-0848-4) contains supplementary material, which is available to authorized users.

## Background

Colorectal cancer (CRC) is the third most common malignant cancer worldwide [1–3]. The mortality of early and mid-term colorectal cancer has been decreased over the last few decades because of improved early diagnosis and therapeutic strategies [4]. However, the overall survival of late stage CRC remains poor [1, 5]. Colorectal carcinogenesis is a multistep process that involves complex genetic and/epigenetic alterations [1]. There is still a need to identify effective molecular markers and therapeutic targets.

The Ubinuclein-2 (UBN2) gene is located on chromosome 7q34 [6]. The UBN2 protein belongs to the ubinuclein family, which exists in numerous human adult and fetal tissues [7]. UBN2 is widely expressed in tumor tissues and encodes a nuclear protein that interacts with viral and cellular transcription factors [8]. The UBN2 protein is detected in the nuclei of cultured keratinocytes and cells originating from the human epidermis [9]. UBN1, an important paralog gene of UBN2 that encodes a partial ubinuclein polypeptide, was aberrantly expressed in many human cancers, including leukemia, CRC, lung carcinoma, cervical carcinoma, melanoma, as well as pancreatic adenocarcinoma [10]. However, the expression and function of UBN2 in cancers is not known.

Mitogen-activated protein kinases (MAPKs) are serine-threonine kinases that play important roles in cell-proliferation, cell adhesion, angiogenesis, invasion and metastasis [11–13]. MAPKs was composed of three major subfamilies: Ras/MAPK, JNK and p38 kinase [13]. Activation of the Ras/MAPK signaling pathway has been implicated in cell proliferation and differentiation via a series of biological cascade reactions in tumors [14–17]. The current study demonstrated that UBN2 was upregulated in CRC tissues. High expression of UBN2 correlated to aggressive characteristics (e.g. lymph node metastasis and distant metastasis) and poor patient survival. UBN2 may promote proliferation, tumor growth and invasion partially via regulation of the Ras/MAPK signaling pathway.

## Materials and methods

### Bioinformatics analysis

GSE5206, GSE9348 and GSE41568 datasets were download from the GEO database. Gene Set Enrichment Analysis (GSEA) was performed to discover the enrichment of signaling pathways for UBN2. The expression of UBN2 in normal and tumor tissues were analyzed using Oncomine network (https://www.oncomine.org/resource/login.html) and TCGA database (https://cancergenome.nih.gov/). The following screening criteria were used for the Oncomine database: UBN2 expression in tumor tissues was twice as high as normal tissues, and the rank of genes was less than 10% and p value less than 0.05.

### Tissue specimens and cell cultures

Liver metastasis tissues (n = 3), CRC tissues and paired normal mucosal tissues (n = 20) were collected during surgery from CRC patients in Nanfang Hospital (Guangzhou, China) in 2015. The samples were embedded in paraffin in the Department of Pathology, Nanfang Hospital, Southern Medical University, China. Thirty freshly collected colorectal cancer tissues and paired normal mucosal tissue specimens were taken from sites distant to the cancerous lesion from patients with CRC undergoing surgical resection. The samples were frozen and stored in liquid nitrogen for further use. Prior approval was obtained from the Southern Medical University Institutional Board prior to the use of these clinical materials for research. All samples were collected and analyzed after written informed consent was obtained from these patients.

Six human CRC cell lines (LoVo, SW837, SW480, HT29, Caco2, and RKO) and one normal intestinal epithelium cell line (FHC) were originally purchased from the American Type Culture Collection (Manassas, VA, USA). The cells were cultured in RPMI-1640 (Gibco, Grand Island, NY, USA) medium containing 10% fetal bovine serum (FBS; Gibco, Grand Island, NY, USA) cells at 37 °C in 5% CO2.

### Immunohistochemistry

Immunohistochemistry (IHC) and scoring were performed as previously described [18]. Two observers reviewed and independently scored the proportion of positively stained tumor cells and staining intensity. The proportion of positive tumor cells was scored as follows: 0 (no positive tumor cells), 1 (< 25% positive tumor cells), 2 (25–50% positive tumor cells), 3 (50–75% positive tumor cells) and 4 (75–100% positive tumor cells). The grading of staining intensity was evaluated using the following criteria: 0 (no staining); 1 (weak staining = light yellow), 2 (moderate staining = yellow brown), and 3 (strong staining = brown). The staining index (SI) was calculated by multiplying staining intensity score and the proportion of positive tumor cells. UBN2 expression was scored as 0, 1, 2, 3, 4, 6, 9, or 12 using this method of assessment. Scores ≤ 3 indicated negative UBN2 expression, and scores > 3 were considered positive for UBN2 expression.

### RNA extraction, reverse transcription (RT) and qRT-PCR

TRIzol reagent (Invitrogen, Carlsbad, CA) was used to extract total RNA from all cell lines and tissues. The cDNA was reverse-transcribed according to a protocol (Takara, Guangzhou, China) and diluted five times with ddH2O. qRT-PCR primers were designed using Primer 5.0 software. The following primer sequences were used: UBN2, sense, 5′-TTATATCAACACTGGCACTCTACA-3′; anti-sense, 5′-TTCCGCTTCCGCTTCTTC-3′; and GAPDH, sense, 5′-GGAGCGAGATCCCTCCAAAAT-3′; anti-sense, 5′-GGCTGTTGTCATACTTCTCATGG-3′. The housekeeping gene GAPDH was used as an interval reference to normalize the data to the geometric mean and calculated using the 2^−ΔΔCT^ method. Each reaction was performed in triplicate.

### Western blotting

Western blotting was performed as previously described [18]. Briefly, equal amounts of protein were separated using electrophoresis on a 10.5% sodium dodecyl sulfate polyacrylamide gel and electro-transferred from the gel to a nitrocellulose membrane. Membrane were blocked with a 5% milk solution in Tris-buffered saline with Tween (TBST) for 1 h and incubated with primary antibodies against KRAS (Proteintech, 12063-1-AP), ERK1/2 (Cell Signaling Technology, #9102), phospho-ERK1/2 (Cell Signaling Technology, #4370), Rac1/2/3 (Cell Signaling Technology, #2465), p21 (Bioworld Technology, #BS6561), p27 (Bioworld Technology, #BS4838), CyclinD1 (Bioworld Technology, BS1741), and UBN2 (ABclone Biotech, A10516). Anti-α-tubulin (Sigma, T9026) was used as an internal loading control.

### Plasmids and transfection

The inhibitor and negative control of UBN2 were purchased from RiboBio (Guangzhou, China). Lipofectamine 2000 reagent (Invitrogen, Carlsbad, CA) was used to transfect the siRNA into CRC cells according to the manufacturer’s instructions. A human shRNA sequence (CCAGTTGGCTCAAGGATAA) specific to UBN2 was cloned into pLenti-U6-puro (ViGene Biosciences, Rockville, MD, USA) to generate pLenti-UBN2-shRNA. Lentiviral products were purchased from VigeneBio (ViGene Biosciences, Rockville, MD, USA) and were transduced according to the manufacturer’s instructions. Stable cell lines expressing shUBN2 were selected using 1 µg/mL puromycin for 10 days.

### Immunofluorescence

Cells were plated on coverslips and fixed with paraformaldehyde (4% w:v). After being rinsed in PBS, the cells were blocked with 0.1% Triton X-100 containing 1% bovine serum albumin in PBS for 1 h. Cells were incubated with a primary antibody against F-actin and a rhodamine-conjugated or FITC-conjugated goat antibody against rabbit IgG (Jackson Immuno Research Laboratories). Coverslips were counterstained with DAPI and imaged using a confocal laser-scanning microscope (Olympus FV1000). Data were processed using Adobe Photoshop 7.0 software.

### In vitro proliferation experiment

1 × 10^3^ cells were seeded into 96-well plates for 24 h. MTT (20 μL of 5 g/L; Sigma-Aldrich, MO, USA) was added into each well and incubated for 4 h at 37 °C. The medium was removed, and 150 μL of DMSO (Sigma Aldrich, MO, USA) was added into each well. The absorption spectrum was detected at 570 nm. For colony formation assays, 200 cells were seeded in a 6-well plate with RPMI-1640 medium containing 10% FBS. Two weeks later, the colonies were fixed using 4% paraformaldehyde for 30 min and stained with hematoxylin. Only colonies containing greater than 50 cells were counted. For the soft agar colony formation assay, 200 cells were seeded in a 6-well plate with soft agar and were incubated for 2 weeks. Only colonies containing greater than 50 cells were counted. Three independent experiments were performed.

### In vitro invasion assay

For the wound healing assays, cells were seed (2 × 10^5^ cells/well) in a 6-well plate and incubated for 24 h. A 10 μL sterile pipette tip was used to create a wound in a straight line. Wound healing was imaged daily.

For the transwell migration assay, cells were serum-starved for 24 h and 1 × 10^5^ cells were plated into the upper chamber of a polycarbonate transwell filter chamber coated with Matrigel (BD). After incubation for 24 h, cells inside the chamber were removed using cotton swabs. The migrated cells on the lower membrane surface were fixed in 1% paraformaldehyde and stained with hematoxylin. Migrated cells were counted under a microscope (10 random 100 X fields per well). Three independent experiments were performed, and the data are presented as the mean ± SEM.

For the three-dimensional culture assay, 24-well culture plates were coated with Matrigel. Cells (1 × 10^5^ cells per well) were seeded incubated for 1 week. A total of five randomly selected fields were chosen to observe the number of invading cells under a microscope.

### Tumorigenesis in nude mice

4–6-week-old Balb/C athymic nude mice (nu/nu) (18–20 g in weight) were obtained from the Animal Center of Southern Medical University, Guangzhou, China. SW837 cells expressed with shUBN2 or scramble control shRNA (2 × 10^6^, n = 7 for each group) were subcutaneously injected into the hind limbs of mice. The Use Committee for Animal Care approved all protocols. Tumor size was measured by a slide caliper and tumor volume was determined as 0.44 × A × B^2^, where A indicates tumor base diameter in one direction, and B indicates the corresponding perpendicular value. Mice were euthanized at 30th day. Tumors were fixed, and 4 µm sections were cut. Hematoxylin and eosin (HE) staining was performed according to standard protocols. Sections were further under stained using an antibody against Ki-67.

### Orthotopic mouse metastatic model

4–6-week-old and Balb/C athymic nude mice (nu/nu) (18–20 g in weight) were anesthetized and underwent surgical orthotopic implantation of the CRC cell lines. A total of 2 × 10^6^ cells in 100 μL PBS were injected into the cecum wall of immunodeficient mice (n = 5 for each group). Mice were euthanized 2 months after surgery, and individual organs were excised. The numbers of gross metastatic foci were determined using a dissection microscope. All the mice used in this study were housed under specific pathogen-free conditions. All animal experiments were performed in accordance with standard procedures and approved by the institutional Use Committee for Animal Care.

### Statistical analysis

Student’s *t* test assuming two-tailed distributions was used to calculate the statistical significance between two groups (GraphPad Prism 6). One-way ANOVO and two-way ANOVA (Tukey’s multiple comparisons test) were used to analyze the statistical significance between multiple groups (GraphPad Prism 6). The Chi square test was used to analyse the correlations between UBN2 expression and clinical pathological parameters using SPSS Statistics software (IBM). Kaplan–Meier analysis was used to evaluate the overall survival (OS) and disease-free survival (DFS). p < 0.05 was considered significant. Kaplan–Meier survival curves were plotted for patients with low versus high UBN2 expression. The mean of UBN2 expression data in the datasets was used as a cut-off. Statistical testing was performed using the log-rank test.

## Results

### UBN2 is upregulated in CRC tissues

**Fig. 1:**
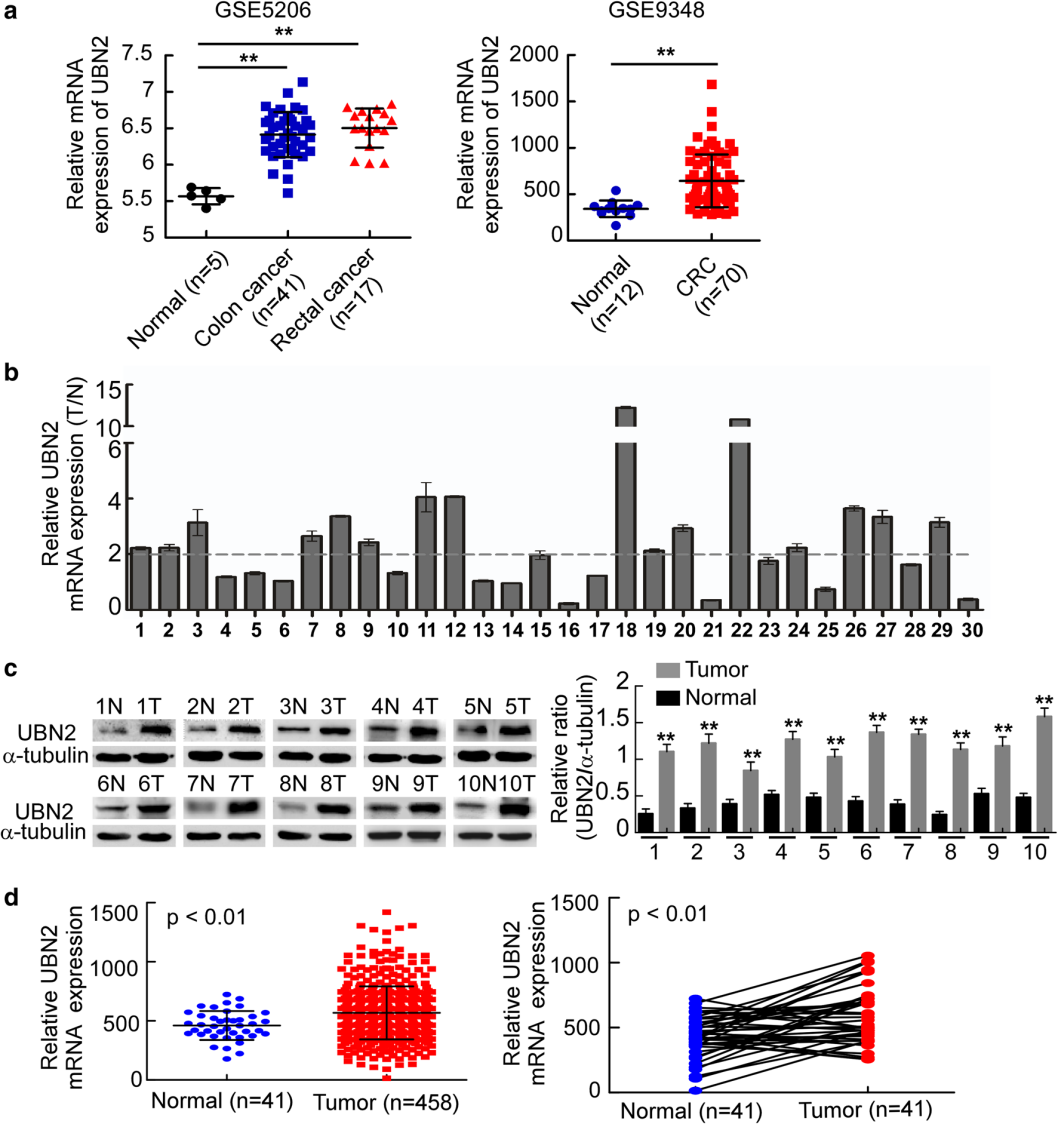
UBN2 is upregulated in CRC tissues. **a** The expression of UBN2 analyzed using Oncomine database (GSE5206 and GSE9348). **b** Expression of UBN2 mRNA in the 30 pairs of CRC specimens. **c** Protein expression of UBN2 in 10 pairs of CRC specimens as detected by Western blotting. The right graph shows the quantification of the Western blotting. **d** Expression of UBN2 mRNA in TCGA CRC database (458 tumor and 41 normal tissues) (left). The right panel shows the mRNA expression of UBN2 in 41 paired tissues (Normal and Tumor) from the TCGA database. Error bars represent mean ± SD from three independent experiments

Analysis using Oncomine demonstrated that UBN2 is upregulated in CRC samples compared with normal tissues (Fig. 1a). qRT–PCR revealed that the tumor/normal (T/N) ratio of UBN2 mRNA levels was higher than 1.5-fold in 77% (23/30) of the tumors, with the highest increase of 24-fold (Fig. 1b). Western blot revealed a significant upregulation of UBN2 protein in all 10 CRC tissues (T) compared to adjacent normal intestine epithelial tissues (N) (Fig. 1c). Analyses using the TCGA CRC database revealed that UBN2 is significantly upregulated in CRC (Fig. 1d, p < 0.01).

### Increased UBN2 expression is associated with CRC progression and poor prognosis

**Fig. 2:**
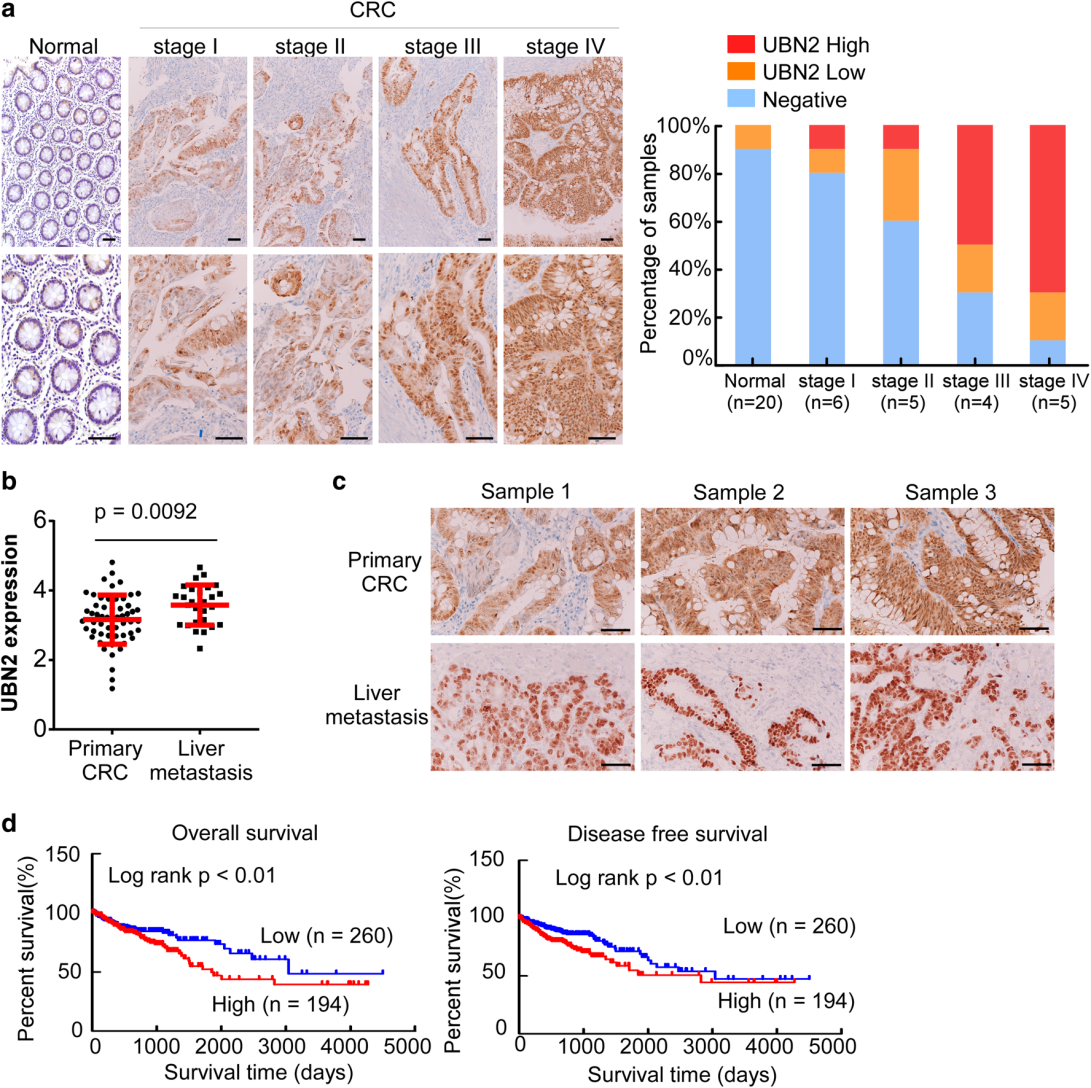
Increased UBN2 expression is associated with CRC progression and poor prognosis. **a** Representative images of UBN2 expression in normal intestinal epithelium and CRC specimens (n = 20) examined by IHC. Scale bars: 50 µm. Histograms represent the statistics of UBN2 expression. **b** The expression of UBN2 in primary CRC and liver metastasis analyzed using Oncomine database (Tsuji colorectal, p < 0.01). **c** Representative images of UBN2 expression in liver metastasis by IHC (n = 3). Scale bars, 50 µm. **d** Effect of UBN2′s expression level on overall survival and disease-free survival by using Kaplan–Meier analyses

The Chi square test analysis showed that there were no correlations between UBN2 expression and age, gender, or T classification (Table 1). However, high UBN2 expression was significantly correlated with advanced clinical stage (p = 0.044), lymph node metastasis (p = 0.037) and distant metastasis (p = 0.025) (Table 1). IHC staining in 20 paired tissues confirmed that the expression of UBN2 is significantly higher in CRC than that in adjacent normal tissues (Fig. 2a). In addition, the expression levels of UBN2 are positively correlated with advanced clinical stages (Fig. 2a). Moreover, analysis using Oncomine showed that the expression of UBN2 is significantly higher in liver metastasis than that in primary CRC tissues (Fig. 2b, p < 0.01), which was further confirmed by IHC staining (Fig. 2c). Furthermore, Kaplan–Meier survival analysis demonstrated that patients with higher UBN2 exhibited significantly poorer overall survival (Fig. 2d, p < 0.01) and disease-free survival (Fig. 2d, p < 0.01).

**Table 1:** Relationship between UBN2 expression and CRC clinicopathological parameters

Characteristics	UBN2 expression		χ^2^ value	*P* value
	Low	High		
Age				
< 60	67	61	0.567	0.539
≥ 60	162	168		
Gender				
Male	124	118	0.315	0.574
Female	105	111		
Stage				
I	38	42	8.101	0.044
II	104	75		
III	56	74		
IV	31	38		
T classification				
T1	7	6	2.53	0.47
T2	37	38		
T3	161	150		
T4	24	35		
N classification				
N0	144	124	4.353	0.037
N1–N2	85	105		
M classification				
M0	180	159	5.007	0.025
M1	49	70

### Downregulation of UBN2 represses human CRC cell proliferation and tumorigenesis

**Fig. 3:**
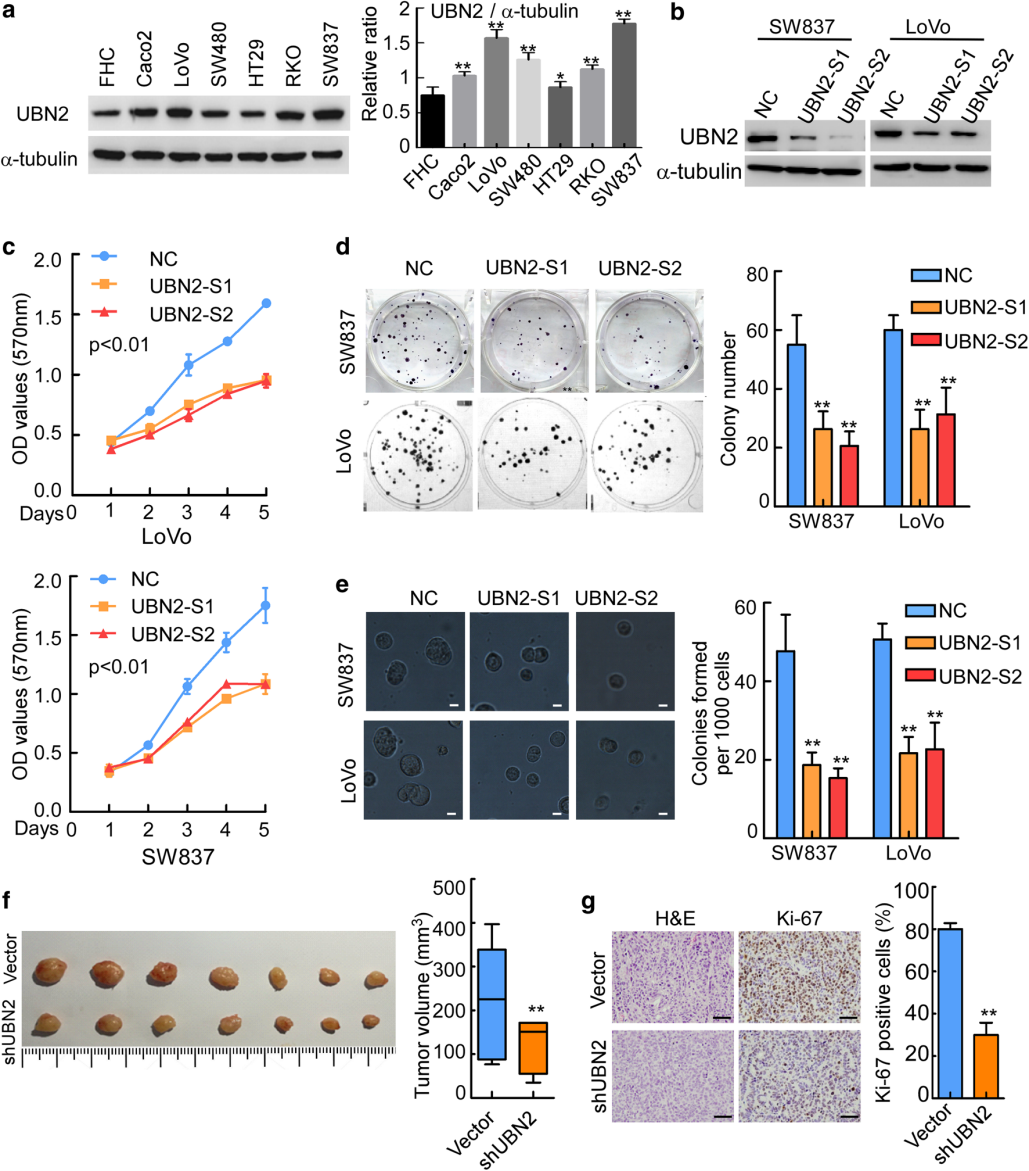
Downregulation of UBN2 represses human CRC cells proliferation and tumorigenesis. **a** The protein expression of UBN2 in CRC cell lines (α-tubulin as an internal reference). **b** Western blot was performed in SW837 and LoVo cells. SW837 and LoVo cells were transfected with vector (NC) or inhibitors (UBN2-S1, UBN2-S2) of UBN2. **c**–**e** Cell proliferation was determined by using MTT (left, factorial analysis, p < 0.01) (**c**), colony formation assays (p < 0.01) (**d**), and soft agar assay (p < 0.01) (**e**). Scale bars: 20 µm. **f**, **g**. SW837/vector and SW837/shUBN2 cells (2 × 10^6^) were injected subcutaneously into nude mice (n = 7). Tumors were collected and measured after the mice were sacrificed (**f**). Lines within boxes and whiskers represent medians and extreme, respectively. All tissues taken from nude mice were subjected to H&E or immunohistochemical staining (Ki-67). Histogram analysis of the percentage of Ki-67-positive cells (**g**). Scale bars: 50 µm. Error bars represent mean ± SD from three independent experiments. **p < 0.01

Western blotting analysis revealed that all six CRC cell lines investigated, including Caco2, LoVo, SW480, HT29, RKO, and SW837 cells, exhibited varying levels of UBN2 expression. Notably, UBN2 expression was relatively lower in normal human colorectal mucous epithelial FHC cells and the less aggressive cell lines (HT29 and SW480) than that in more aggressive cell lines (SW837 and LoVo) (Fig. 3a). To study the molecular of UBN2 in CRC, UBN2 was silenced using siRNAs specific to human UBN2 (Fig. 3b). MTT assay showed that knockdown of UBN2 significantly decreased the growth rate of SW837 and LoVo cells compared with negative control (NC) cells (Fig. 3c, p < 0.01). Colony formation assay (Fig. 3d, p < 0.01) and soft agar assay (Fig. 3e, p < 0.01) displayed that knockdown of UBN2 dramatically reduced colony size and colony numbers compared with controlled cells. To investigate the effects of UBN2 on tumorigenesis in vivo, UBN2 were stably knockdown in SW837 cells (SW837/ShUBN2) using specific UBN2 shRNA hairpins. Subcutaneous tumorigenesis assay demonstrated that knockdown of UBN2 significantly inhibited tumor growth in nude mice (Fig. 3f, p < 0.01). IHC staining revealed that knockdown of UBN2 dramatically decreased the Ki-67 index in SW837 tumors (Fig. 3g, p < 0.01).

### UBN2 inhibition reduces CRC cell migration and invasion and tumor metastasis in vitro and in vivo

**Fig. 4:**
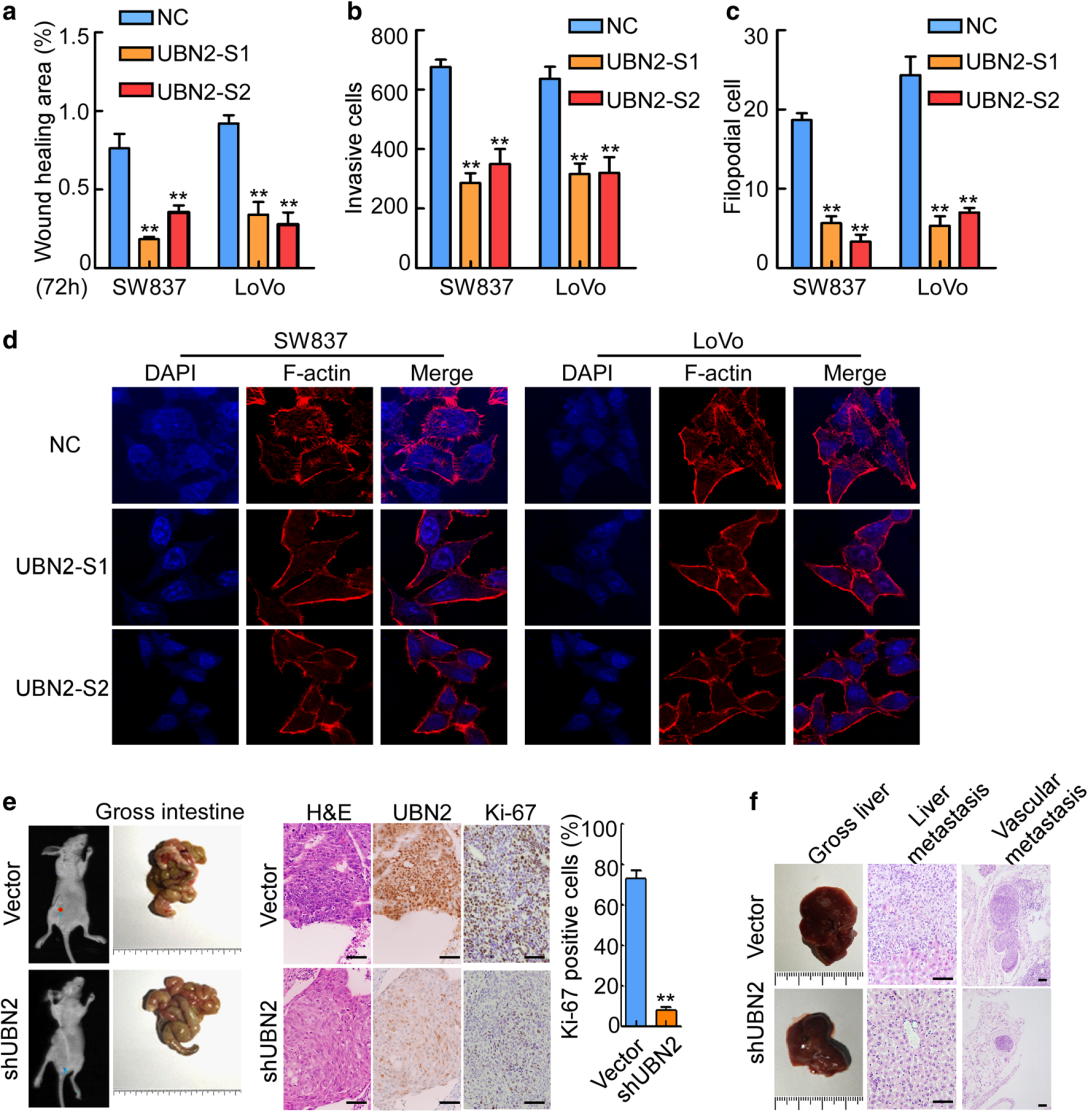
UBN2 inhibition reduces CRC cell migration and invasion and tumor metastasis in vitro and in vivo. **a** Cell migration was determined by using wound-healing assays. Histograms represent the percentage of wound-healing area in 72 h. **b** Quantification of the numbers of migrated cells by transwell migration assay. Histograms represent the number of invasive cells. **c** Three-dimensional morphology assay. Histograms represent the average number of filopodia formed by each cell sphere. **d** F-actin expression and pseudopodia were measured in UBN2-depleted SW837 and LoVo cells. **e** Primary tumors in the intestines formed in mice orthotopically implanted with SW837/shUBN2 and control SW837 cells. H&E staining and IHC staining by using antibodies against UBN2 and Ki-67 were shown. Scale bars: 50 µm. Histograms represent the percentage of Ki-67 positive cells. **f** Representative images of gross specimens and H&E staining of liver and vascular metastatic lesions. Scale bars: 50 µm. Error bars represent mean ± SD from three independent experiments. **p < 0.01

The wound-healing and transwell assays showed that knockdown of UBN2 in SW837 and LoVo cells significantly suppressed cell migration (Fig. 4a and Additional file 1: Fig. S1A, p < 0.01) and invasion (Fig. 4b and Additional file 1: Fig. S1B, p < 0.01). Three-dimensional cultures revealed that cells with knockdown of UBN2 showed less invasive pseudopodia formation than control cells (Fig. 4c and Additional file 1: Fig. S1C, p < 0.01). In addition, immunofluorescence staining revealed that knockdown of UBN2 decreased F-actin expression and reduced pseudopodia formation in CRC cells (Fig. 4d). Orthotopic metastatic assay demonstrated that knockdown of UBN2 suppressed the growth of primary CRC tumors and inhibited the Ki-67 proliferation index (Fig. 4e, p < 0.01). Importantly, UBN2 knockdown dramatically suppressed the average number of micro-metastases in liver and vascular metastasis as determined by H&E staining (Fig. 4f). Taken together, these findings demonstrated that UBN2 promotes migration, invasion and metastasis of CRC cells.

### UBN2 inhibition suppresses CRC proliferation and metastasis via the Ras/MAPK signaling pathway

**Fig. 5:**
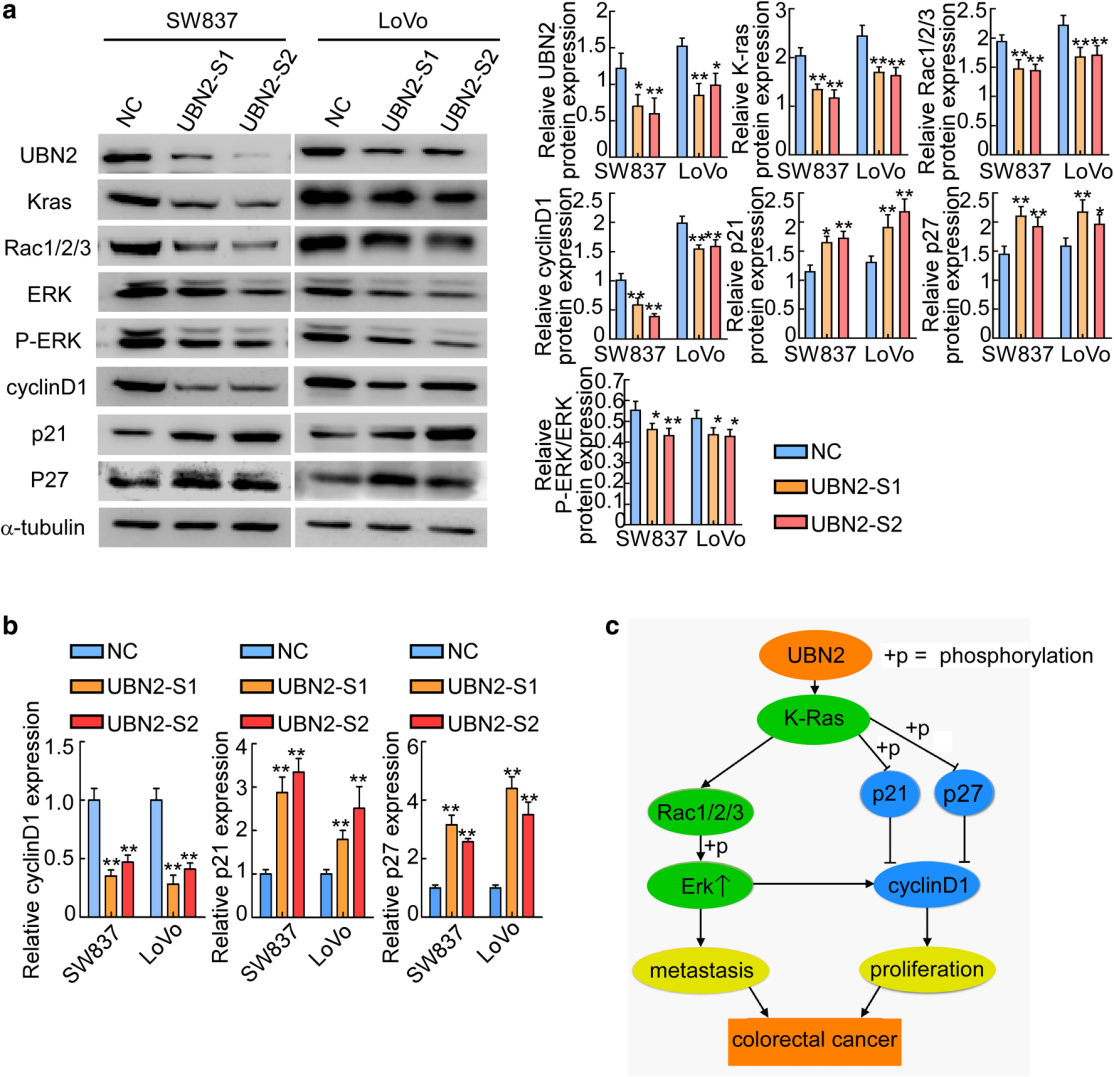
UBN2 inhibition suppresses CRC Proliferation and Metastasis via the Ras/MAPK Signaling Pathway. **a** The expression of UBN2, KRAS, Rac1/2/3, total ERK, phosphorylated ERK, cyclinD1, p21 and p27 in vector-infected (NC) and UBN2 siRNA-infected (UBN2-S1, UBN2-S2) CRC cell lines were detected by Western blotting. **b** Real-time RT-PCR analyses of cyclinD1, p21, and p27 were performed in the indicated CRC cell lines. Error bars represent mean ± SD from 3 independent experiments. **c** A model for UBN2 regulation of tumorigenesis. *p < 0.05, **p < 0.01

GSEA enrichment analysis revealed that the KRAS-up signaling pathway was enriched in the high UBN2 expression group (Additional file 2: Fig. S2, p < 0.01), which suggests a role for UBN2 in the regulation of KRAS signaling. Next, we tested the role of UBN2 on the Ras-MAPK signaling pathway. Interestingly, UBN2 knockdown decreased the expression of KRAS, Rac1/2/3, p-ERK, and cyclinD1, while increased the expression of p21, and p27 (Fig. 5a). In addition, knockdown of UBN2 decreased the expression of cyclinD1 and increased the expression of p21 and p27 at mRNA level (Fig. 5b, p < 0.01).

## Discussion

The ubinuclein family is widely expressed in malignant tissues, including CRC [11]. However, the expression pattern and function of UBN2 during the progression of CRC is not clear. The present study demonstrated that UBN2 was upregulated in CRC tissues. In addition, overexpression of UBN2 notably correlated with the invasive and aggressive features, including advanced clinical stage, lymph node metastasis, distant metastasis, high proliferation index and poor prognosis. These findings suggest that high UBN2 protein expression is an independent prognostic marker to identify patients with poor clinical outcomes.

The high rate of proliferation is the primary cause of rapid tumor growth [19]. The imbalance between positive and negative regulatory factors is an important cause of the unlimited proliferation of tumors [20, 21]. The present study demonstrated that knockdown of UBN2 decreased the proliferation and tumorigenicity of CRC cells, indicating the oncogenic role of UBN2 in CRC.

It was well known that tumor cells undergo a series of pathological events during the process of metastasis, including deep invasion into the intestinal wall, entry into the circulatory or lymph-vascular system, arrest at a remote site, proliferation and the induction of angiogenesis [22]. Our study found that knockdown of endogenous UBN2 notably weakened the migratory and invasive abilities of CRC cells in vitro and in vivo. These results suggested that UBN2 may be involved in metastasis via promoting migration and invasion of tumor cells.

Ras/MAPK signaling pathways are involved in numerous biological processes such as cell proliferation [23–25]. Additionally, activation of the Ras/MAPK pathway promotes cyclinD1 expression and inhibits the p21 and p27 levels [26–28]. Moreover, MAPK signaling pathway is correlated with cell adhesion, angiogenesis, invasion, and metastasis in CRC [29, 30]. For example, the Ras/MAPK pathway contributes to the upregulation of vascular endothelial growth factor expression and the induction of angiogenesis in CRC [31]. PKCβII activates CRC cell invasion via Ras/MEK pathway [32]. The MAPK pathway is also used as a molecular target for the detection and treatment of metastatic CRC [33]. The current study showed that UBN2 upregulated KRAS expression and activated Ras/MAPK signaling. It has been reported that either UBN1′s or UBN2′s subunit is mainly responsible for specific recognition and direct binding of H3.3 by the HIRA complex [34]. Two independent HIRA complexes (UBN1-HIRA and UBN2-HIRA) can cooperatively deposit H3.3 to cis-regulatory regions, including activating promoters and activating enhancers in mouse embryonic stem (mES) cells [34]. These data suggested that UBN2 may participate in KRAS gene transcription as a part of histone chaperone. KRAS is frequently mutated or dysregulated in CRC and it is involved in the modulation of downstream effectors Rac/Rho to promote tumor metastasis [35]. KRAS signaling also regulates the cell cycle via by phosphorylation and inhibiting p21 and p27 to relieve cyclinD1 [36, 37] (Fig. 5c).

## Conclusions

The current study demonstrates that UBN2 acts as an oncogene in CRC. UBN2 may be used as a promising biomarker for predicting the prognosis of CRC patients. However, the potential function and specific mechanisms of UBN2 in human CRC should be investigated in detail.

## Additional files

**Additional file 1: Fig. S1.** UBN2 inhibition reduces CRC cell migration and invasion and tumor metastasis in vitro. A. Representative results of wound-healing assays in 0 h, 36 h, and 72 h. Scale bars: 100 μm. B. Cell invasion was determined by using the transwell migration assay. Scale bars: 100 μm. C. Three-dimensional morphology assay. Only cell colonies > 0.1 mm in diameter were counted. Scale bars: 20 μm.

**Additional file 2: Fig. S2.** UBN2 expression is positively correlated with Kras signaling. The Kras-up signaling pathway is enriched in the high UBN2 expression group from the GEO database (GSE41568, n = 155).

## Data Availability

The datasets generated and/or analyzed during the current study are available in the GEO, GSE41568, GSE5206, GSE9348. All data generated or analyzed during this study are included in this published article.

## Abbreviations

CRC: colorectal cancer
TCGA: The Cancer Genome Atlas
MAPK: mitogen-activated protein kinase
MMP9: matrix metallopeptidase 9
RT: reverse transcription
IHC: immunohistochemistry
p21: p21Cip1/WAF1
p27: p27Kip1
