# Supplementary figures and images for: UBN2 promotes tumor progression via the Ras/MAPK pathway and predicts poor prognosis in colorectal cancer
Source: Cancer Cell Int. 2019 May 10;19:126. doi: 10.1186/s12935-019-0848-4 (PMC6511126; doi:10.1186/s12935-019-0848-4)

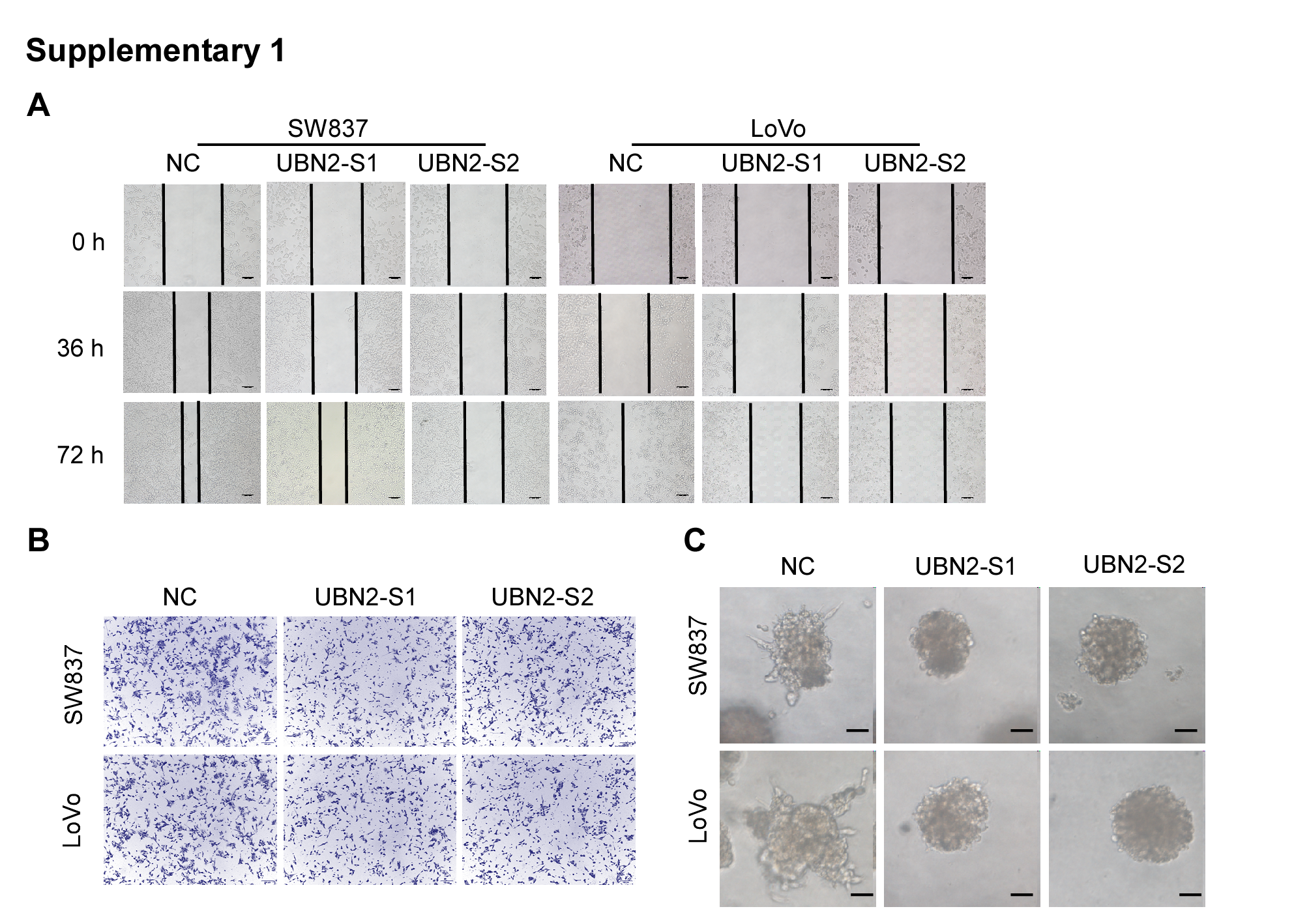

Supplement: Supplementary file 1 — Additional file 1: Fig. S1. UBN2 inhibition reduces CRC cell migration and invasion and tumor metastasis in vitro. A. Representative results of wound-healing assays in 0 h, 36 h, and 72 h. Scale bars: 100 μm. B. Cell invasion was determined by using the transwell migration assay. Scale bars: 100 μm. C. Three-dimensional morphology assay. Only cell colonies > 0.1 mm in diameter were counted. Scale bars: 20 μm. [file 12935_2019_848_MOESM1_ESM.tif]

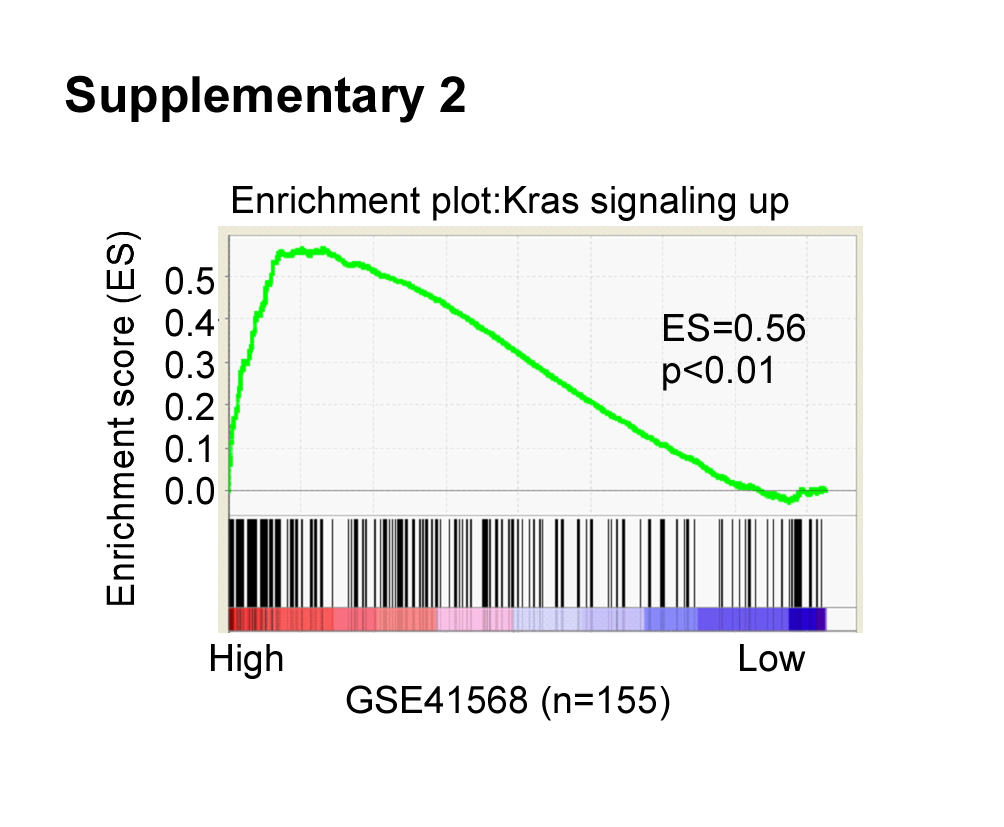

Supplement: Supplementary file 2 — Additional file 2: Fig. S2. UBN2 expression is positively correlated with Kras signaling. The Kras-up signaling pathway is enriched in the high UBN2 expression group from the GEO database (GSE41568, n = 155). [file 12935_2019_848_MOESM2_ESM.tif]
